# Investigation of the effects of salt stress on morphological, physiological, biochemical, antioxidant characteristics, and gene expression responses in pistachio(*Pistacia vera* L.)

**DOI:** 10.1186/s12870-026-08408-x

**Published:** 2026-02-25

**Authors:** Hooman Shirvani, Foad Fatehi, Sara Hejri, Ramesh Katam

**Affiliations:** 1 Department of Agriculture, Payame Noor University, Tehran, Iran; 2 Institute of Agricultural Biotechnology, National Institute for Genetic Engineering and Biotechnology, Tehran, Iran; 3 Department of Plant Biology, Faculty of Biosciences, Kharazmi University, Tehran, Iran; 4 Department of Biological Sciences, Florida A&M University (FAMU), Tallahassee, Florida USA

**Keywords:** Salt stress, pistachio, antioxidant, gene expression

## Abstract

**Background:**

Salt stress is a major limiting factor for agricultural productivity, particularly in arid and semi-arid regions. It adversely impacts plant growth and physiological functions. Understanding the morphological, biochemical, and molecular responses of pistachio to salinity is crucial for developing salt tolerant cultivars.

**Results:**

In this study, UCB-1 pistachio seedlings (*Pistacia vera* L.) were subjected to 0, 100, and 200 mM NaCl for 7 and 21 days to investigate their morphological, physiological, biochemical, and molecular responses to salt stress. Salinity significantly reduced fresh and dry weights of roots and stems, as well as chlorophyll and carotenoid contents. In contrast, proline, soluble carbohydrates, and phenolic compounds increased, indicating adaptive metabolic responses. Salt stress also enhanced the accumulation of reactive oxygen species (ROS) and malondialdehyde (MDA), reflecting oxidative stress. Enzymatic activities of SOD, CAT, APX, and GPX were significantly upregulated under salt stress. Gene expression analysis revealed a marked induction of *NHX1*, *Dehydrin*, *CAT*, and *DREB2* genes, particularly at 200 mM NaCl after 21 days, indicating their pivotal roles in ion homeostasis, osmoprotection, and antioxidant defense.

**Conclusions:**

These findings highlight the complex physiological and molecular mechanisms involved in salt tolerance of pistachio. The activation of antioxidant systems and stress responsive genes contributes to mitigating oxidative damage and maintaining cellular homeostasis. These results provide insights into the adaptive responses of pistachio to salinity and may contribute to breeding efforts aimed at developing salt tolerant cultivars suitable for saline environments.

**Supplementary Information:**

The online version contains supplementary material available at 10.1186/s12870-026-08408-x.

## Background

Soil salinity is one of the most significant abiotic stresses that limits agricultural productivity and sustainability, particularly in arid and semi-arid regions. Factors such as poor irrigation management, climate change, and poor soil drainage exacerbate salt accumulation in these areas. Approximately 20% of irrigated land worldwide is affected by salinity, posing a serious threat to global food security. High concentrations of soluble salts in the soil disrupt plant growth and development through osmotic stress, ion toxicity, and nutrient imbalances. These factors contribute to impair cellular functions and metabolic processes [1, 2], leading to a decrease in plant biomass, yield, and overall vitality. Consequently, understanding plant responses to salinity is essential for developing salt tolerant cultivars and the implementation of sustainable agricultural practices.

Pistachio (*Pistacia vera* L.) is a valuable perennial tree crop that holds significant economic and nutritional importance, especially in countries such as Iran, the United States, and Turkey. Due to its inherent drought and salinity tolerance, pistachio is widely cultivated in semi-arid regions often experience high salinity levels. However, when salinity levels exceed a certain threshold, they can severely impair the growth and yield pistachio trees by disrupting water and nutrient uptake, photosynthesis, and physiological integrity [3]. Research indicates that the estimated threshold for reduction in pistachio yield reduction is 8.65 dS/m in soil and 4.2 dS/m in irrigation water [4].

Salt stress has been shown to reduce root and shoot biomass, leaf dry weight, and stem elongation while increasing leaf succulence and specific leaf area [5]. At the physiological level, salinity inhibits photosynthesis by limiting chlorophyll content, reducing stomatal conductance, and interfering with carbon fixation. It also leads to cellular dehydration due to reduced osmotic potential. Biochemically, plants respond to salt stress by accumulating osmoprotectants such as proline, glycine betaine, and soluble sugars to maintain turgor pressure and stabilize cellular structures. However, prolonged exposure to salt can result in excessive production of reactive oxygen species (ROS), including superoxide anions, hydrogen peroxide, and hydroxyl radicals, which damage lipids, proteins, nucleic acids, and membranes [6]. The accumulation of malondialdehyde (MDA), a marker of lipid peroxidation, is often used to assess oxidative damage under stress conditions.

To mitigate the damage caused by reactive oxygen species (ROS), plants activate an elaborate antioxidant defense system comprising both enzymatic and non-enzymatic components. Enzymatic antioxidants such as superoxide dismutase (SOD), catalase (CAT), and ascorbate peroxidase (APX) play a pivotal role in detoxifying ROS, while non-enzymatic molecules like phenolics, flavonoids, and carotenoids serve as secondary scavengers [7, 8]. In pistachio, antioxidant responses vary markedly among different genetic backgrounds and between plant tissues such as leaves and roots. These responses are also strongly influenced by both the severity and duration of salt exposure. Under salinity stress, more tolerant root systems typically activate a more efficient antioxidant machinery and maintain superior ion compartmentalization, while sensitive backgrounds show weaker defense responses and are more prone to oxidative damage [9].

Recent advances in genomics, transcriptomics, and bioinformatics have led to a deeper understanding of salt tolerance mechanisms in pistachio. Genome sequencing and transcriptomic analysis have identified key gene families, such as cytochrome P450s and chitinases, that are expanded in pistachio and contribute to stress resilience [10]. Furthermore, expression of genes involved in ion transport (e.g., *NHX*, *HKT*, and *SOS1*), osmolyte biosynthesis, stress related transcription factors (e.g., *DREB*, *WRKY*, *NAC*), and hormonal signaling pathways (e.g., ABA and jasmonic acid) are upregulated in response to salinity [11, 12]. Physiological adaptations such as vacuolar Na⁺ sequestration, increased suberization of root endodermis, and improved root-to-shoot ion exclusion capacity also contribute to salt tolerance, particularly in genotypes such as UCB1 [13].

Pistachio’s relatively high tolerance to salinity, but the increasing salinization of arable land driven by climate change and unsustainable agricultural practices underscores the urgent need to investigate crop responses to stress. A comprehensive understanding of the morphological, physiological, biochemical, and molecular responses of pistachio to salinity can aid in identifying salt tolerant genotypes and unraveling the underlying regulatory networks. The present study aims to systematically examine the impact of salt stress on multiple biological levels in pistachios, focusing on morphological changes, physiological traits, oxidative responses, and expression of key stress related genes. By integrating these parameters, this study provides a comprehensive evaluation of pistachio adaptive responses to salinity, enhancing our understanding of the physiological, biochemical, and molecular mechanisms involved, and contributes to efforts aimed at developing crops with improved tolerance to salt affected environments.

## Results

### Gene expression

**Fig. 1:**
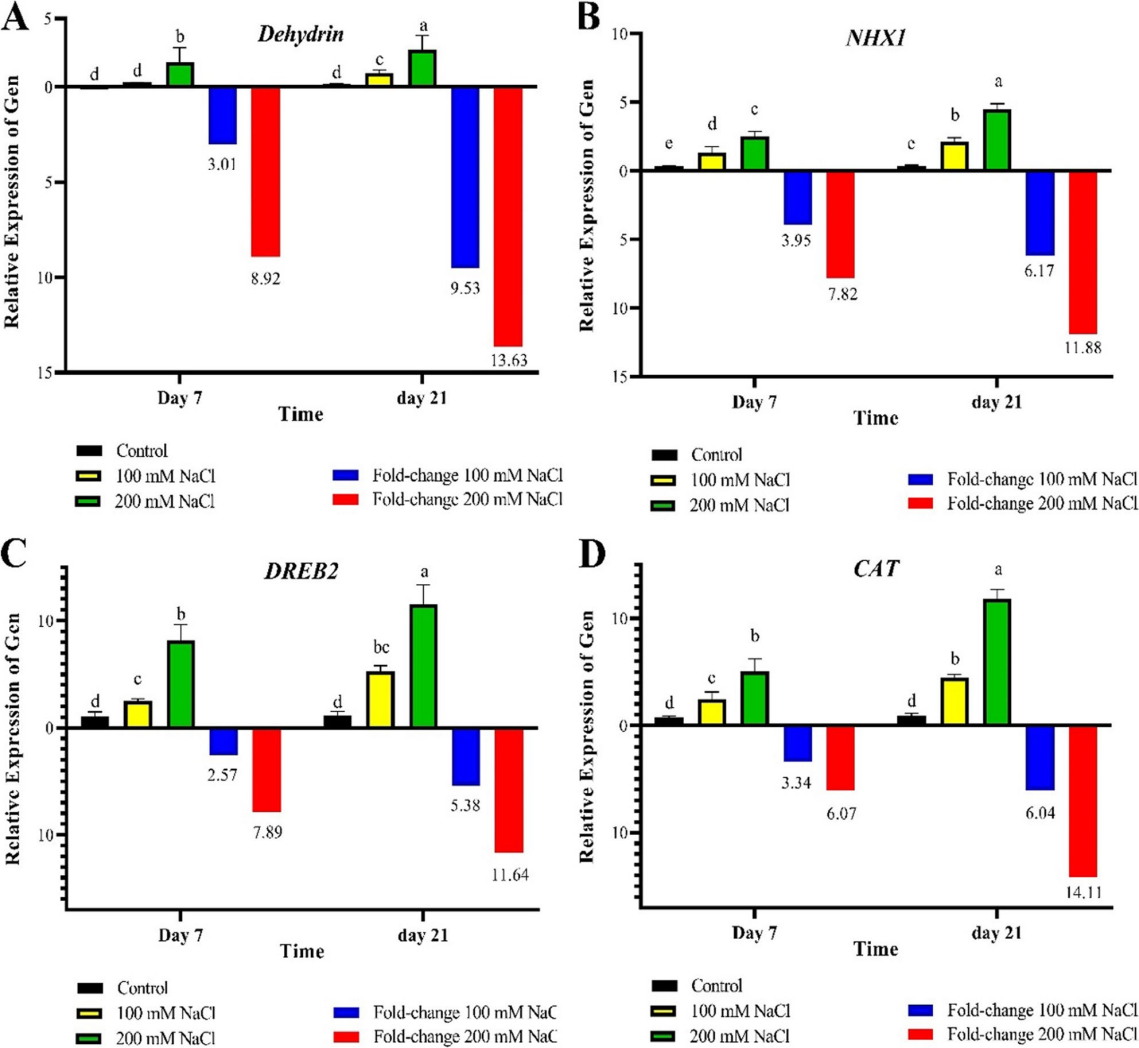
Comparison of mean and fold-change of the expression of the genes under study at different durations and salt stress levels. Fold-change values were calculated relative to the control treatment after normalization using the reference gene

Analysis of variance (ANOVA) studies showed that both salinity stress and the duration of exposure to salinity had significant effects on the expression levels of *NHX1*, *Dehydrin*, *CAT*, and *DREB2* genes at the 1% probability level (*P* < 0.01) (Supplementary Table S1). Stress notably affected the expression of all analyzed genes, with the most pronounced effects observed for *CAT* and *DREB2*, exhibiting mean square values of 4.318 and 4.294, respectively. The exposure time affects the plant’s transcriptional responses to salinity stress. The duration of salinity exposure significantly influenced gene expression across all target genes at the 1% significance level. The *Dehydrin* and *CAT* genes, with mean squares of 0.153 and 2.712 respectively, demonstrated the strongest responses to stress duration. The interaction between salinity stress and its duration also significantly affected gene expression. While this interaction was significant at the 5% level for *Dehydrin*, notable interactive effects were also recorded for *NHX1* and *CAT*, with mean squares of 0.108 and 1.073, respectively. The significant interaction between stress intensity and duration underscores the complexity of gene regulation under abiotic stress conditions. At both 7 and 21 days after the application of salt stress, the expression of *Dehydrin* gene showed a significant increase compared to the control treatment. The highest expression was observed in the 200 mM NaCl treatment on day 21, showing an approximately 14 fold-change compared to the control. A significant increase was also observed at 100 mM, although it was lower than the severe treatment (Fig. 1-A).

The expression of the *NHX1* gene significantly increased under salt stress. On day 21, in the 200 mM treatment, the expression increased by about 12-fold. Significant increases were also observed in other treatments, especially in those with longer exposure times (Fig. 1-B). The *DREB2* gene expression increased under both salt conditions, particularly in the 200 mM treatment on day 21, where its expression reached 12 fold-change. Increases were also observed on day 7, but were less pronounced compared to day 21 (Fig. 1-C). The *CAT* gene exhibited high expression in response to salt stress. The most significant increase was noted in the 200 mM treatment on day 21, achieving a 14 fold-change compared to the control. This trend was also evident on day 7 and at lower concentrations, though with less intensity (Fig. 1-D).

### Measured traits

The effect of salt stress was significant across all the morphological, antioxidant, biochemical, and physiological variables with the analysis of variance at the 1% probability for most variables. Additionally, the duration of salt stress application had a significant impact on other variables except for the stem fresh weight and stem dry weight. The interaction between salt stress and its duration did not show a significant effect on chlorophyll b, hydrogen peroxide, stem fresh weight, and stem dry weight, however, significant differences were observed for other traits (Supplementary Table S2).

### Morphological traits

**Fig. 2:**
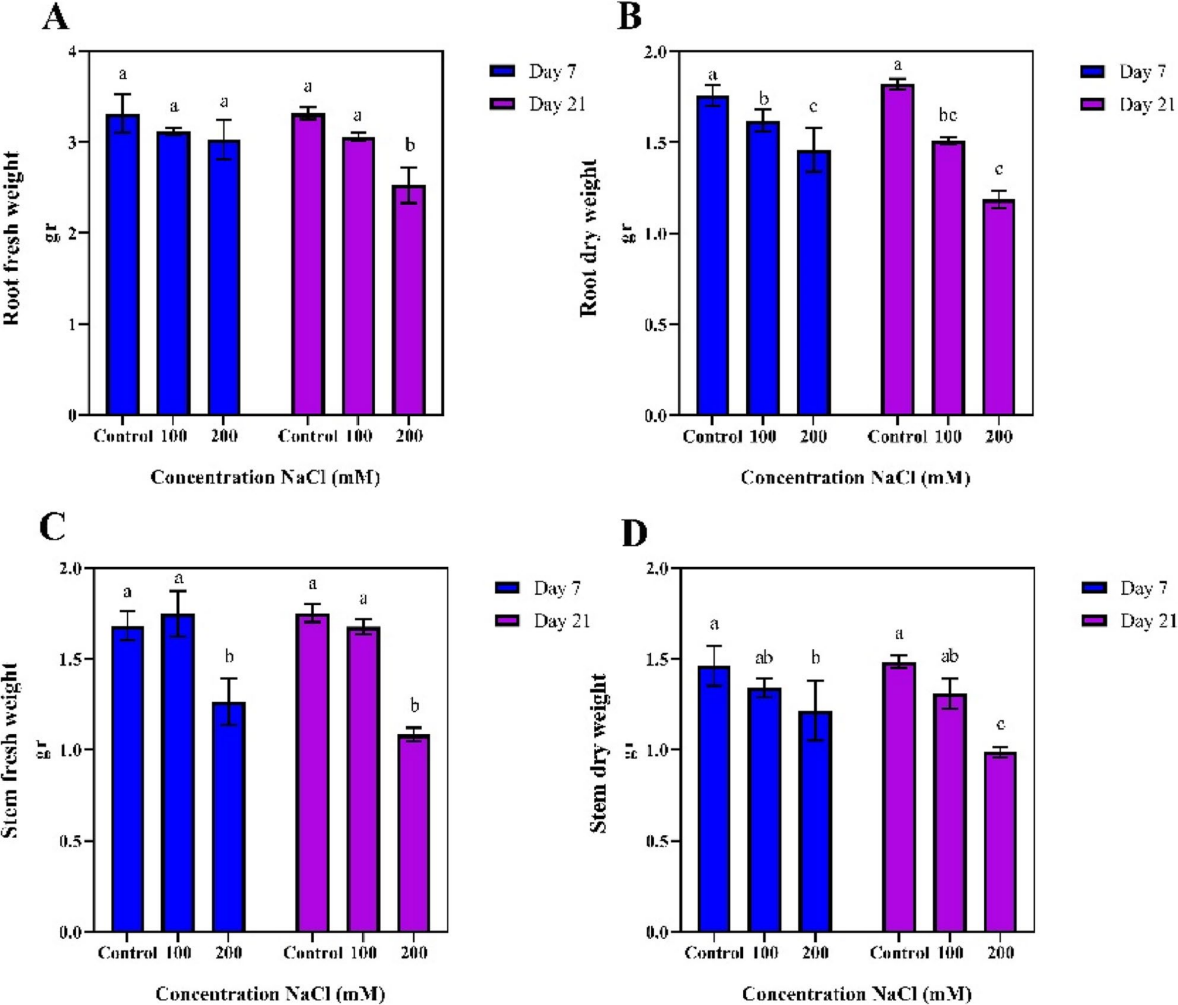
Comparison of mean values of morphological traits at different durations and salinity stress levels

The highest root fresh weight was observed in the control and 100 mM NaCl treatments at both 7 and 21 days (3.312, 3.314, and 3.311 g, respectively). Conversely, the lowest fresh root weight was recorded for the 200 mM NaCl treatment at day 21 (2.5 g) (Fig. 2-A).

The highest root dry weight was also recorded in the control treatment at days 7 and 21 (1.61 and 1.82 g, respectively). The lowest dry root weight was observed in the 200 mM NaCl treatment at days 7 and 21 (1.46 and 1.18 g) (Fig. 2-B).

For the stem, the highest stem fresh weight was found in the control and 100 mM NaCl treatments at days 7 and 21 (1.68, 1.75, and 1.74 g, respectively). In contrast, the lowest fresh stem weight was recorded for the 200 mM NaCl treatment at days 7 and 21 (1.23 and 1.08 g) (Fig. 2-C). The highest stem dry weight was observed in the control treatment at days 7 and 21 with the values of 1.46 and 1.48 g. Meanwhile, the lowest dry stem weight observed in the 200 mM NaCl treatment at day 21 at 0.98 g (Fig. 2-D).

### Physiological traits

**Fig. 3:**
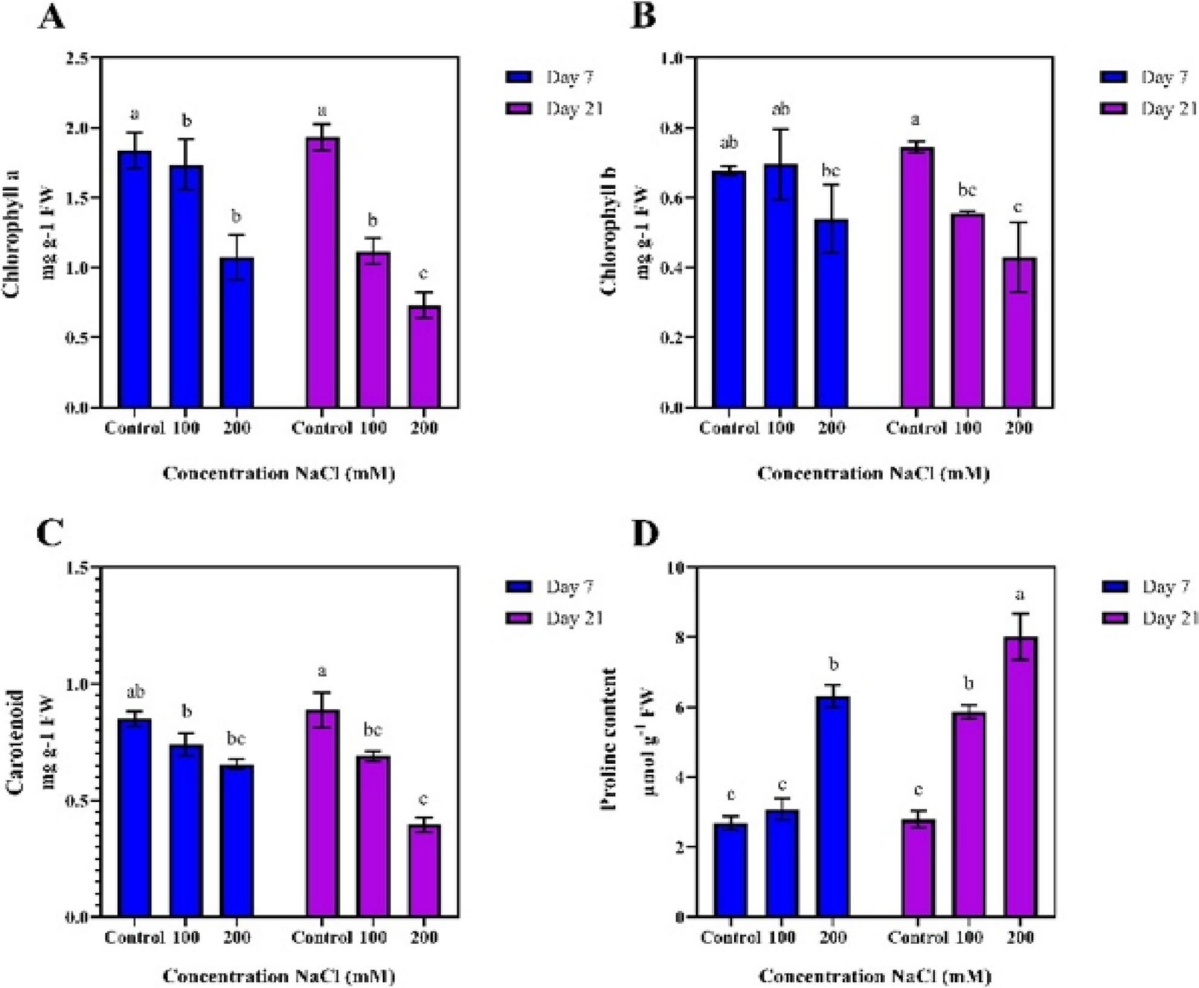
Comparison of mean values of physiological traits at different durations and salinity stress levels

The highest chlorophyll a content was observed in the control treatment on days 7 and 21 (1.838 and 1.932 mg/g). In contrast, the lowest value was recorded in the 200 mM NaCl treatment on day 21 (0.821 mg/g) (Fig. 3-A). Similarly, chlorophyll a, chlorophyll b decreased under salt stress. The highest chlorophyll b content was observed in the control treatment on day 21 (0.767 mg/g), while the lowest content was recorded in the 200 mM NaCl treatment on the same day (0.410 mg/g) (Fig. 3-B). Carotenoids, which are important antioxidants in photosynthesis, also decreased under salt stress. The highest carotenoid content was observed in the control treatment on day 21 (0.846 mg/g), whereas the lowest value was recorded in the 200 mM NaCl treatment on the same day (0.416 mg/g) (Fig. 3-C).

In contrast, proline, as an important osmolyte in osmotic pressure regulation, significantly increased in response to salt stress. The highest proline content was recorded in the 200 mM NaCl treatment on day 21 (8.01 µmol/g), while the lowest values were observed in the control treatment on days 7 and 21, and in the 100 mM NaCl treatment on day 7 (2.69, 2.79, and 3.10 µmol/g) (Fig. 3-D).

### Biochemical traits

**Fig. 4:**
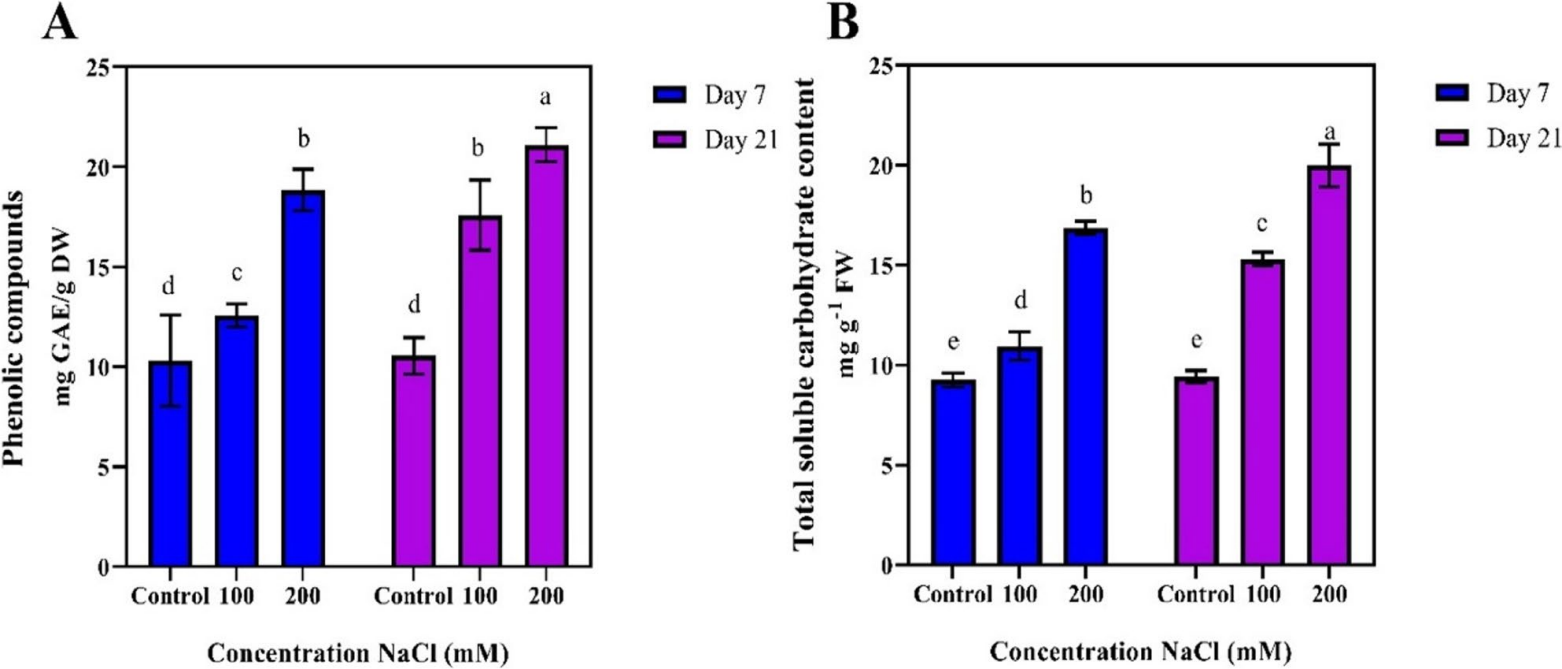
Comparison of mean values of total phenolic compounds and total soluble carbohydrates under different durations and salinity stress levels

On day 7, the highest content of phenolic compounds was observed in the 200 mM NaCl treatment, measuring 21.1 mg of gallic acid per gram. In contrast, the control treatment and the 100 mM NaCl treatment showed decreased phenolic content, with values of 10.13 and 10.55 mg of gallic acid per gram, respectively. However, by day 21, phenolic compounds in the 200 mM NaCl treatment significantly increased, surpassing the other treatments (Fig. 4-A).

The lowest amount of soluble carbohydrates was observed in the control treatment with measurements of 9.25 and 9.41 mg/g on on days 7 and 21 respectively. In contrast, as the NaCl increased, the soluble carbohydrate content also rose. By day 21, the highest soluble carbohydrate level was recorded in the 200 mM NaCl treatment, reaching 19.98 mg/g, which represented a significant increase compared to the other treatments, especially when compared to day 7 (Fig. 4-B).

### Oxidative traits

**Fig. 5:**
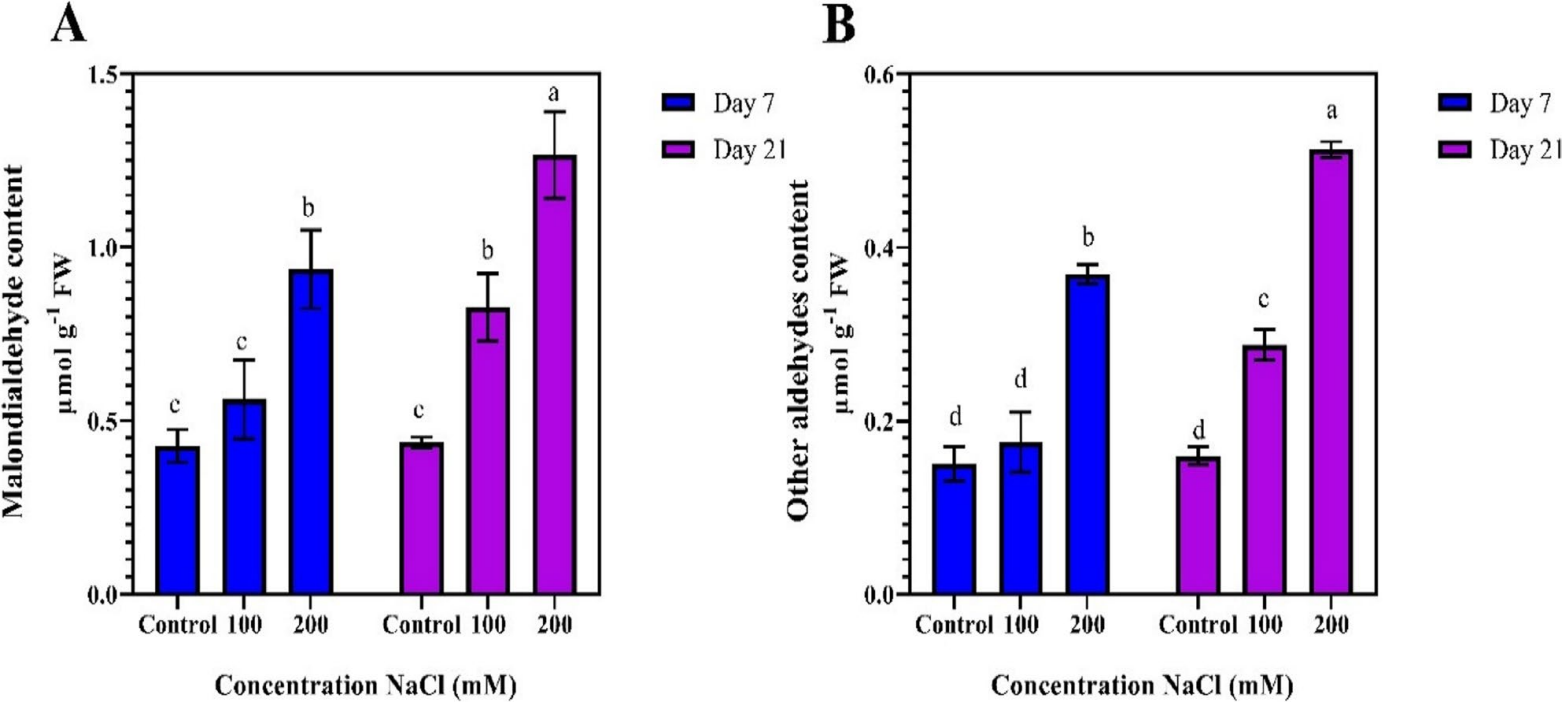
Comparison of mean values of oxidative traits at different durations and salinity stress levels

On days 7 and 21, the control treatment showed the lowest malondialdehyde (MDA) content, measuring 0.427 µmol/g and 0.437 µmol/g, respectively. With an increase in NaCl concentration to 100 and 200 mM, MDA levels significantly increased. On day 21, the highest MDA content was observed in the 200 mM NaCl treatment (1.266 µmol/g), which was significantly higher than the 100 mM NaCl and control treatments (Fig. 5-A). Similarly, on days 7 and 21, the lowest levels of other aldehydes were observed in the control treatment measuring 0.15 and 0.16 µmol/g. With increasing NaCl concentration, the levels of these aldehydes also increased accordingly. On day 21, the highest levels of other aldehydes were observed in the 200 mM NaCl treatment (0.513 µmol/g), indicating the increasing impact of salt stress on the production of oxidative aldehydes over time (Fig. 5-B). On day 21, the highest levels of other aldehydes were detected in the 200 mM NaCl treatment, at 0.513 µmol/g. This trend indicates that the impact of salt stress on the production of oxidative aldehydes increases over time (Fig. 5-B).

### Antioxidant traits

**Fig. 6:**
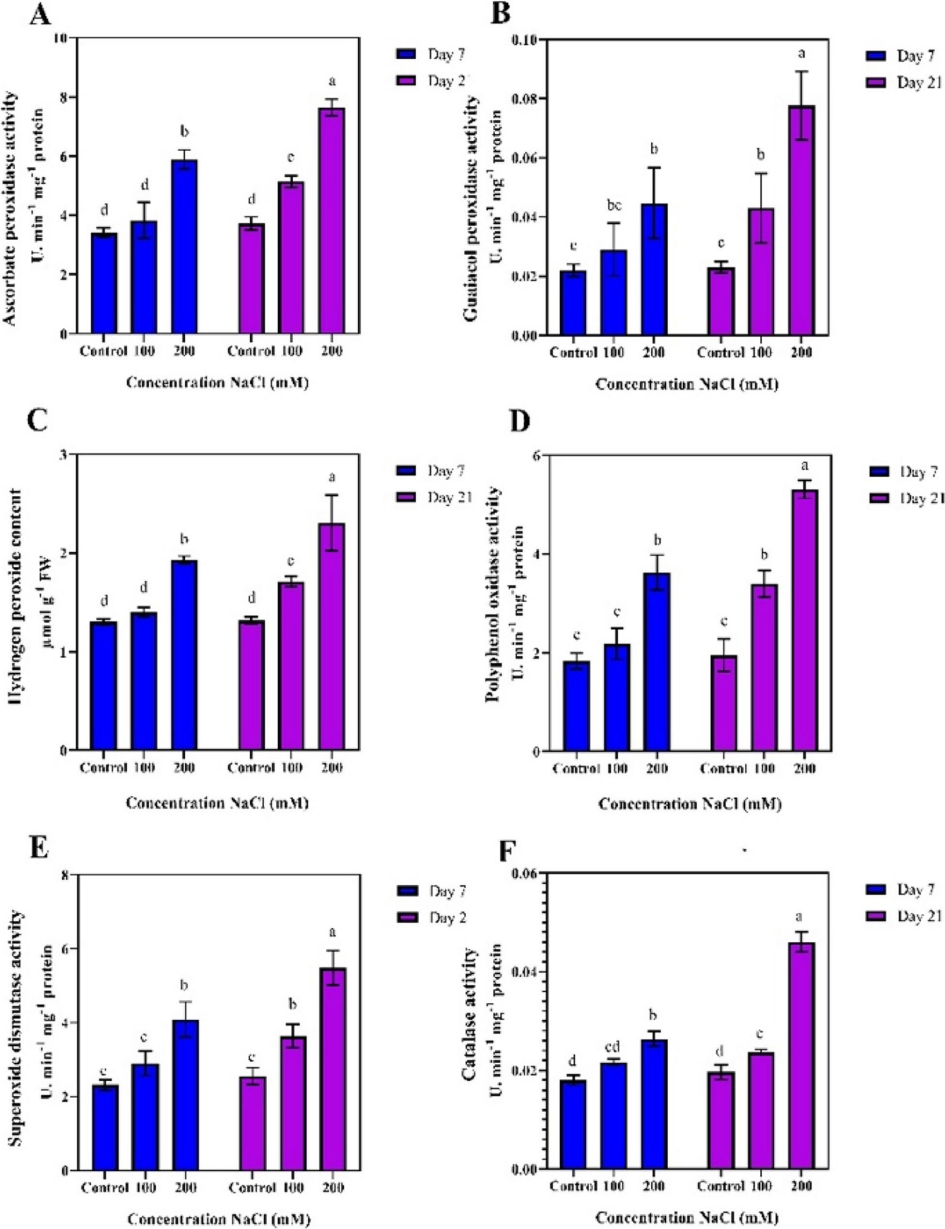
Comparison of mean values of antioxidant traits at different durations and salinity stress levels

The ascorbate peroxidase (APX) enzyme activity significantly increased under saline stress conditions (100 and 200 mM NaCl). This increase was more pronounced particularly on day 21 compared to day 7. Notably, on day 21, the highest APX activity was recorded in the 200 mM NaCl treatment, with a measurement of 7.64 µmol/min/mg. In contrast, the lowest APX activity was recorded in the control treatments on days 7 and 21 and in the 100 mM NaCl treatment on day 7 (3.41, 3.72, and 3.83 µmol) (Fig. 6-A). Similarly, the activity of guaiacol peroxidase (GPX) also increased significantly in high salinity treatments, particularly at 200 mM NaCl treatment, similar to APX. The highest GPX activity was recorded on day 21 (0.78 µmol/min/mg). The lowest GPX activity was observed in the control treatment on days 7 and 21 (0.22 and 0.23 µmol/min/mg) (Fig. 6-B).

Hydrogen peroxide (H₂O₂) content also increased significantly with increasing salinity stress, particularly at the 200 mM NaCl level. On day 21, H₂O₂ levels were significantly higher than those recorded in the control treatment and 100 mM NaCl treatment on days 7 reaching 2.304 µmol/g, compared to 1.30, 1.31, and 1.04 µmol/g, respectively for the control and 100 mM NaCl treatments. (Fig. 6-C).

Polyphenol oxidase (PPO) activity significantly increased with increasing salinity, especially at the 200 mM NaCl, with the highest activity recorded on day 21 (5.30 µmol/min/mg). The lowest PPO activity was observed in the control treatments on days 7 and 21 and in the 100 mM NaCl treatment on day 7 (1.83, 1.95, and 2.17 µmol/min/mg) (Fig. 6-D). Superoxide dismutase (SOD) activity significantly increased under salinity stress. The highest SOD activity was recorded in the 200 mM NaCl treatment on day 21 (5.47 µmol/min/mg), while the lowest activity was observed in the control treatments on days 7 and 21 and the 100 mM NaCl treatment on day 7 (2.31, 2.55, and 2.89 µmol/min/mg) (Fig. 6-E). Catalase (CAT) activity significantly increased in response to salinity stress. The highest CAT activity was recorded at the 200 mM NaCl level on day 21 (0.46 µmol/min/mg), while the lowest CAT activity was observed in the control treatments on days 7 and 21 (0.18 and 0.20 µmol/min/mg) (Fig. 6-F).

## Discussion

Environmental stresses, such as drought and salinity, are significant factors that greatly impact the physiological and biochemical traits of plants. Particularly in economic trees like pistachio, these stresses can pose serious challenges to their production and performance. Numerous studies have been conducted in this field, showing that plants exposed to these stresses undergo significant changes in their physiological and biochemical structures.

Analysis of variance (ANOVA) revealed a significant and pronounced effect of salt stress on the expression of genes involved in defense responses and environmental stress tolerance. These findings suggest that the duration of salt stress exposure directly influences gene expression patterns and can modulate plant metabolic regulatory mechanisms in response to salinity. Furthermore, the results demonstrate that both the intensity and duration of salt stress act synergistically to regulate gene expression. Such observations contribute to a deeper understanding of the molecular mechanisms underlying salt stress tolerance in plants and hold valuable implications for breeding programs aimed at developing salt tolerant cultivars. Previous research has shown that NAC1 expression increases in both salt sensitive and salt tolerant pistachio genotypes under salinity, whereas SOS1 is specifically upregulated in the tolerant genotype (eBR1). These findings indicate that, unlike the broader stress responsive role of NAC1, SOS1 may function as a genotype dependent regulator of ion transport and contribute more directly to salinity tolerance [14]. Afshar and Abbaspour [15], demonstrated that *arbuscular mycorrhizal* fungi (*Rhizophagus irregularis*) can alleviate salinity stress in *Pistacia vera* by enhancing biomass, reducing oxidative damage, and upregulating both enzymatic and non-enzymatic antioxidant systems. The expression of stress related genes such as *Cu/Zn-SOD*, *Fe-SOD*, *Mn-SOD*, and *GR* was significantly increased in AMF-inoculated plants, highlighting the role of mycorrhizal symbiosis in improving salt tolerance through transcriptional and physiological regulation.

The *Dehydrin* gene is primarily expressed under water deficit conditions, suggesting that salt stress leads to cellular dehydration, which triggers its expression. The time dependent expression pattern of this gene highlights its progressive role in adaptive mechanisms, particularly under prolonged stress conditions where sustained cellular protection is necessary. Overall, the data indicate that *Dehydrin* functions as an early and gradually responsive gene to salinity stress, predominantly influenced by the duration of exposure rather than the severity of the stress, with limited interaction between these two factors. The *NHX1* gene, a key component in ion homeostasis, facilitates the sequestration of sodium ions into vacuoles, thus mitigating ion toxicity. Its increased expression under saline conditions reflects the activation of adaptive responses aimed at maintaining ionic balance and reducing sodium induced damage. Additionally, *DREB2*, a transcription factor involved in regulating genetic responses to abiotic stresses such as drought and salinity, also exhibited enhanced expression, indicating the activation of stress related signaling pathways that may lead to the upregulation of additional defense related genes. Furthermore, the increased expression of the antioxidant enzyme *CAT* in response to salt stress signifies the activation of the antioxidant defense system to counteract the elevated levels of reactive oxygen species (ROS) commonly produced under stress conditions. Collectively, the results of this study suggest that pistachio plants enhance their tolerance to salinity by coordinating the expression of genes associated with osmolyte metabolism, ion balance, transcriptional regulation, and antioxidant defense systems.

Adaptation to salt stress in *pistachio* is likely linked to the expansion of cytochrome P450 and chitinase gene families. Population genomics study further traced pistachio domestication to approximately 8000 years ago, identifying genes related to tree and seed size under artificial selection, and highlighting the role of the jasmonic acid biosynthesis pathway in salinity tolerance [10]. Notably, expression of stress responsive genes including *SOS1*, *CCX2*, *SKOR*, *NRT2.4*, *PHO1*, and *PIP2.4* was positively regulated by AMF colonization, indicating that arbuscular mycorrhizal symbiosis enhances ionic homeostasis and osmoprotection under salt stress in *Pistacia vera* [16].

The analysis of variance for morphological, physiological, biochemical, and antioxidant traits indicated that salinity stress and the duration of exposure significantly affected various plant characteristics in pistachio. Specifically, the interaction between salinity intensity and duration highlighted the complexity of the plant’s response mechanisms to environmental changes and its ability to modulate different reactions under stress conditions. These findings suggest that the length of salinity exposure of salinity can influence both the type and intensity of plant responses, ultimately influencing its adaptability to adverse environmental conditions.

Increasing salinity significantly reduced the fresh weight of both roots and stems, primarily due to disruptions in water and nutrient uptake caused by impaired root and stem function. The reduction in fresh weight was more on day 21, indicating the cumulative effects of salinity on root and stem structure and function. Decline in root fresh weight was observed at 200 mM NaCl treatment, attributed to increased osmotic pressure and the disruption of moisture uptake by the roots and stems. Furthermore, salinity led to a significant reduction in the dry weight of both roots and stems, indicating cellular structural damage, decreased organic matter production, and a reduced ability of the plant to store energy and cope with environmental stress. The decrease in dry weight of the roots and stems may be linked to disruptions in cell division and a reduction in metabolic activities, which, in turn, limit plant growth and reduce its capacity to accumulate nutrients. Overall, the reduction in fresh and dry weights of the roots and stems under salt stress highlights the negative impact of salinity on plant growth and performance, primarily due to disruptions in water uptake and reduced photosynthetic activity, ultimately limiting plant growth and development under saline conditions. Similar morphological and pomological variations observations were made earlier on 44 *Pistacia atlantica* accessions, where a significant difference were found among the genotypes, with high variability in traits such as leaf length (87.52–157.80 mm) and nut dimensions (nut length: 5.49–8.23 mm, nut thickness: 4.39–6.42 mm, they further noted a strong correlation were observed between 100 nut weight and other traits. characteristics, including accessions 1 and 7 of subsp. cabulica [17].

Salinity significantly reduced the chlorophyll a content, particularly on day 21, when the cumulative effects of long term salinity stress became more evident. Our studies reveal that this reduction is primarily attributed to the degradation of the photosynthetic structure of chloroplasts and disruption of chlorophyll synthesis processes. The decrease in chlorophyll a, which was more pronounced at a salinity level of 200 mM NaCl, may lead to a reduction in photosynthetic efficiency and, consequently, a decrease in biomass production. Previous evaluations of pistachio rootstocks under salinity stress have reported declines in leaf area, chlorophyll content, and stomatal conductance, accompanied by increased electrolyte leakage and enhanced antioxidant activity. Based on these physiological responses, the studied rootstocks were recommended for cultivation under moderate salinity conditions, highlighting the importance of selecting genotypes with inherently stronger stress mitigation capacities [18]. High salinity has also been shown to disrupt the photosynthetic machinery by degrading photosynthesis related proteins and reducing chlorophyll stability, thereby limiting the plant’s capacity to recover from stress induced damage. In a comparative study examining four pistachio rootstocks exposed to 0, 75, and 150 mM NaCl, the authors reported genotype specific responses to salinity, indicating that different rootstocks employ distinct physiological and biochemical strategies to cope with increasing salt levels [19].

A significant reduction in chlorophyll b under high salinity treatments indicates damage to the light absorption and energy transfer systems in the plant. Moreover, the decrease in carotenoids due to salinity could suggest a disruption in light absorption and a reduction in the plant’s capacity to cope with ROS. This reduction primarily reflects the limitation of the plant’s defense mechanisms against oxidative stress induced by salinity. The increased proline content, particularly under severe salinity treatment, indicates the plant’s effort to mitigate the osmotic stress effects and protect cells from structural and metabolic damage. Our results indicate that proline accumulation plays a crucial role in the adaptation of pistachio plants to long term salinity stress, contributing to the maintenance of photosynthetic performance and osmotic balance under saline conditions.

Increased phenolic content, particularly at higher salinity levels, especially at a concentration of 200 mM NaCl, was significant and indicative of the activation of the plant’s defense mechanisms in response to salinity stress (Ref). Phenolic compounds act as effective antioxidants that neutralize ROS, and their increase under salinity stress reflects the defensive role of these compounds. The increase in soluble carbohydrate content indicates their role as energy sources and compatible osmolytes, which help regulate osmotic pressure and enhance the plant’s tolerance to salinity stress. This increase was particularly more evident on day 21, further highlighting the protective role of carbohydrates under saline stress conditions. Hajiboland et al. [20] demonstrated that pistachio is a salt tolerant species, with mild salinity maintaining compatible solutes such as proline and soluble sugars in leaves and roots. Under high salinity, the synthesis of proteins was reduced, and carbohydrates were preferentially allocated to roots, leading to growth inhibition. At low salinity levels, sodium concentration in leaves did not increase significantly, but higher salt concentrations altered the K: Na and Ca: Na ratios. Previous research on pistachio rootstocks has shown that salinity levels above 100 mM markedly suppress plant growth, with notable reductions in biomass and photosynthetic performance [21].

In this study, salinity stress has been shown to increase oxidative damage in pistachio, particularly at higher NaCl concentrations, with a significant increase in MDA at the 200 mM concentration. MDA levels, known as a marker for oxidative stress, increased significantly due to salinity stress, confirming the occurrence of cellular damage and the plant’s response to adverse environmental conditions. Notably, there was a significant rise in MDA levels at the 200 mM concentration. MDA is a recognized marker for oxidative stress, and its increase indicates cellular damage, reflecting the plant’s response to adverse environmental conditions. As the NaCl concentration increases, so do the levels of MDA and other aldehydes, which suggest oxidative damage due to salinity stress. In a study conducted by Mirfattahi et al. [5], the effects of salinity on different pistachio genotypes (*Pistacia vera* Akbari, Ghazvini, and *P. vera* × *P. atlantica*) were investigated in a soilless culture the researchers found that salt stress (0, 50, 100, and 150 mM NaCl) reduced plant growth but increased specific leaf area (SLA). The study observed increased malondialdehyde (MDA) and proline accumulation, with Ghazvini exhibiting the highest MDA concentration and lowest cell membrane stability. The results indicated that leaf abscission, SLA, leaf succulence, MDA concentration, and photosynthetic pigment degradation are useful parameters for screening salt tolerance.

Salt stress leads to elevated production of ROS, particularly H₂O₂, reflecting the oxidative pressure experienced by pistachio plants under saline conditions. In response, the plant activates a network of antioxidant defenses to mitigate cellular damage. APX and CAT play central roles in detoxifying H₂O₂, preventing its accumulation and maintaining redox balance. Similarly, SOD converts superoxide radicals into H₂O₂, which is subsequently removed by APX and CAT, demonstrating a coordinated defense system. GPX and polyphenol oxidase further contribute by neutralizing ROS and regulating polyphenol metabolism. The observed increases in the activities of these enzymes under salinity highlight the plant’s dynamic adjustment to oxidative stress, strengthening cellular protection and ensuring survival under challenging environmental conditions. In previous studies, Mirabi et al. [22] studied the response of two pistachio genotypes, Badami-e-Zarand (BZ) and Badami-e-Sefid (BS), to varying NaCl concentrations. Both genotypes showed increased proline levels under stress; however, BS faced greater oxidative stress, reflected in impaired photosynthetic performance along with reduced chlorophyll and carotenoid content. In contrast, BZ demonstrated enhanced activities of antioxidant enzymes (APX, CAT, and GPX) under high salinity and a significant boost in SOD activity at moderate salinity levels, highlighting its resilience compared to BS. Further studies on two interspecific pistachio hybrids, Arota 1 and Arota 2, along with their parent rootstocks (*P. atlantica* and *P. integerrima*), under varying saline conditions (0, 100, and 200 mM NaCl) found that Arota hybrids, particularly Arota 2, exhibited notable salinity tolerance. This was evidenced by reduced sodium levels, higher potassium content, enhanced antioxidant enzyme activities, and lower lipid peroxidation [23].

## Conclusion

This study demonstrated that salt stress significantly affects the morphological, physiological, biochemical, and molecular characteristics of pistachio cv. UCB-1. The observed reduction in fresh and dry weights of roots and stems, as well as chlorophyll and carotenoid contents, indicates the plant’s response to salt stress. Concurrently, increases in proline, soluble carbohydrates, and phenolic compounds suggest adaptive strategies that help pistachio cope with NaCl induced oxidative stress. Moreover, enhanced activities of antioxidant enzymes, including SOD, CAT, APX, and GPX, along with upregulation of key genes such as *NHX1*, *Dehydrin*, *CAT*, and *DREB2*, highlight the importance of these mechanisms in mitigating oxidative damage and maintaining ion homeostasis. The significant interaction between the intensity and duration of salt stress underscores the complexity of the regulatory networks involved. These findings have clear practical implications. They provide valuable insights for breeding programs and genetic engineering efforts aimed at developing salt tolerant pistachio cultivars. Furthermore, understanding these physiological and molecular responses can inform field management practices, such as irrigation and salinity mitigation strategies, to enhance pistachio productivity under saline conditions. Future research could focus on identifying additional genes involved in salt tolerance and evaluating the performance of these mechanisms under field conditions, ensuring that laboratory findings translate into agronomically meaningful outcomes.

## Materials and methods

### Experimental conditions

Six month-old UCB-1 pistachio rootstocks procured from Tooba Tissue Culture Company, Iran. The UCB-1 variety is a hybrid pistachio rootstock, developed at the University of California, USA, through the controlled cross pollination of Atlantic and Integrima varieties. These rootstocks were planted in 50 × 50 cm pots containing a 1:1 mixture of sand and clay. The experiment was conducted in a greenhouse under a 16-hour light / 8-hour dark photoperiod, with day/night temperatures of 28 °C/18°C and a relative humidity of 70%. At the time of planting, the pistachio rootstocks were in the juvenile growth phase, characterized by vigorous vegetative growth and rapid root and shoot development. This developmental stage is associated with high growth rates and intense metabolic activity, which results in increased sensitivity to environmental stressors [24]. The plants were cultivated in a factorial experiment based on a completely randomized design with three biological replicates under greenhouse conditions. The experimental factors included three salinity levels (0 [control], 100, and 200 mM NaCl) as the first factor, and two durations of salt stress exposure (7 and 21 days) as the second factor. The plants were cultivated under greenhouse conditions in a factorial experiment based on a completely randomized design. The experimental factors included three salinity levels (0 [control], 100, and 200 mM NaCl) as the first factor, and two durations of salt stress exposure (7 and 21 days) as the second factor. For the assessment of morphological, physiological, and biochemical traits, all three independent biological replicates were used (Each biological replicate consisted of a single, independent UCB-1 pistachio seedling). However, for gene expression analysis, two biological replicates were selected from these three, and each biological replicate was measured with two technical replicates to ensure precise quantification of relative gene expression levels.

### Salt stress treatment

Salt stress was applied using three NaCl concentrations: 0 (control), 100, and 200 mM. Irrigation was performed from the top, and soil moisture was maintained at field capacity. The required amount of NaCl for each concentration was calculated based on soil analysis and the desired molarity, ensuring precise and consistent control of salinity throughout the experiment. The calculation was performed as follows:

$${\mathrm C}_{\mathrm{mg}/\mathrm{kg}}\;\approx\;1000\;{\mathrm C}_{\mathrm{mol}/\mathrm L}\;{\mathrm M}_{\mathrm g/\mathrm{mol}}$$

$${11688}_{mg/kg}\;\approx\;1000\;\times\;0.2_{mol/L}\;\times\;58.{44}_{g/mol}$$

$${\mathrm C}_{\mathrm{mg}/\mathrm{kg}}\;\approx\;1_{\mathrm L/\mathrm{kg}}\;\times\;{\mathrm C}_{\mathrm{mg}/\mathrm L}$$

$${11688}_{\mathrm{mg}/\mathrm{kg}}\;\approx\;1_{\mathrm L/\mathrm{kg}}\;\times\;{11688}_{\mathrm{mg}/\mathrm L}$$

After conducting a soil analysis, pots were irrigated with Hoagland’s nutrient solution for four weeks to ensure adequate nutrient availability. Salinity treatments were then applied using NaCl concentrations of 0 (control), 100, and 200 mM. Each pot received a fixed volume of 2 L of salt solution every four days. Soil salinity was monitored before each irrigation using a conductivity meter (EC meter, model 5TE, Decagon Devices, Pullman, WA, USA). To maintain target salinity levels and prevent salt accumulation, drainage water from each pot was reused to adjust the salinity of subsequent irrigations, and leaching with 1 L of deionized water for 10 min was performed when necessary to ensure uniform experimental conditions [25].

### Gene expression analysis

In this study, we analyzed the expression of *NHX1*, *Dehydrin*, *CAT*, and *DREB2* genes to investigate the molecular responses of the UBC-1 pistachio genotype to salt stress. RNA extraction was performed using the RNA X Plus kit. The quantity, quality, and concentration of RNA were assessed using a NanoDrop spectrophotometer (model 2000 C, Thermo Fisher Scientific, USA). The quality of the extracted RNA was further confirmed through 1% agarose gel electrophoresis.

The RNA samples were treated with the DNase I kit (Thermo Fisher Scientific, USA). cDNA synthesis was carried out using the Reverse Transcription Kit (CinnaGen, Iran). In the first step, the required components were added to a cDNA production tube and incubated at 55 °C for 60 min. Following this, the samples were incubated at 95 °C for 5 min in a hot water bath. Finally, the tubes were placed on ice and stored at -80 °C for further use. For real-time PCR analysis, the Amplicon 2X Real-Time PCR Master Mix was used. To optimize the conditions for each primer pair, a mix of all treatments and replicates of the synthesized cDNA was prepared. Various dilution factors were tested for each primer pair to determine the optimal concentration. Once the optimal concentrations of primers and cDNA were identified, Real-Time PCR was performed using the Applied Biosystems thermal cycler (AB1). The sequences of the primers used, along with the reference gene, are listed in Table 1. The relative expression levels of the examined genes were then calculated using the 2^−∆∆CT^ relationship based on the obtained melting temperature for each primer [26]. Ct values were first normalized against the expression of a reference gene (housekeeping gene, *NADH*) to account for variations in cDNA quantity. Fold-change values were then calculated relative to the control treatment (0 mM NaCl) at each corresponding time point, such that the expression level of the control was set as 1. This approach allowed for the assessment of induction or repression of gene expression in response to different salt stress levels and durations.

**Table 1:** Sequence characteristics and Melting temperature of primers used in gene expression analysis

Primer name	Sequence (5’-3’)	Temperature (°C)	Band size (bp)	Accession numbers
*NADH-F*	GGAGACTCAAATGGTGGATA	56	216	XM_031413090
*NADH-R*	ACCTGCTAGTGGAGGAAGAC			
*NHX1-F*	GTGCGGTATCTATGGCACTTG	59	123	XM_031411879
*NHX1-R*	CGAACACCACTGTGCTGAAAA			
*CAT-F*	CAGATACTACGTGCGGTGG	58	97	XM_031398761
*CAT-R*	TGGGAGATGCTGTGAGAGAA			
*Dehydrin-F*	ATGGATATGGCGTAGAGAAG	55	156	Y07600
*Dehydrin-R*	TACTTGGGATCTCATTCACC			
*DREB2-F*	CCTGCGTGTCAAATGCTGAG	60	252	XM_031395858
*DREB2-R*	AGGCCGCAAGTTCACCATAG			

### Measurement of physiological parameters

To measure the fresh weights of shoots and roots, the aerial parts of the plants were excised using scissors. The fresh weight of the roots (after surface drying with paper towels) and shoots were recorded using a digital balance with an accuracy of 0.001 g. For dry weight determination, the samples were placed in an oven at 105 °C for 6 h, after which the dry weight was measured with the same precision. Chlorophyll and carotenoid contents were measured according to the method of Lichtenthaler and Welburn [27], . Approximately 25 mg of leaf tissue from wheat was ground in liquid nitrogen and homogenized in 2 mL of 96% ethanol in darkness. The mixture was then thoroughly shaken and centrifuged at 10,000 rpm for 10 min at 4 °C. The supernatant was transferred to a microplate, and absorbance was measured at 663, 646, and 470 nm using an ELISA reader (BioTek Powerwave XS2). The concentrations of chlorophyll a, chlorophyll b, total chlorophyll, and carotenoids were calculated using the following equations:

$$\mathrm{Chl}\;\mathrm a\;=12.21\;\left({\mathrm A}_{663}\right)\;-\;2.81\;\left({\mathrm A}_{646}\right)$$

$$\mathrm{Chl}\;\mathrm b\;=20.13\;\left({\mathrm A}_{646}\right)\;-\;5.1\;\left({\mathrm A}_{663}\right)$$

$$\mathrm{Ch}1\;\mathrm T\;=\mathrm{Ch}1\;\mathrm a\;+\;\mathrm{Chl}\;\mathrm b$$

$$\mathrm{Car}\;=(1000\;{\mathrm A}_{470}\;-3.27\;\left[\mathrm{Ch}1\;\mathrm a\right]-\;104\;\left[\mathrm{Ch}1\;\mathrm b\right]/227$$

### Measurement of biochemical parameters

Total soluble proteins and antioxidant enzymes were measured using the published protocols [28]. To prepare 200 mL of extraction buffer, 2.428 g of Tris and 0.2 g of PVP were weighed and dissolved in 40 mL of distilled water. The pH of the solution was then adjusted to 8 using hydrochloric acid, and the final volume was brought up to 200 mL, and used for extraction fo soluble proteins and antioxidants. Polyphenol oxidase activity (PPO) was measured according to the method of Kar and Mishra [29], . Catalase activity (CAT) was assessed based on the method of Sinha [30] with slight modifications. The quantitative concentration of ascorbate peroxidase (APX) was determined using the method of Nacano and Asada [31], . Guaiacol peroxidase (GPX) activity was evaluated according to Lin and Kao [32], . The hydrogen peroxide (H_2_O_2_) concentration was measured following the method of Velikova et al. [33]. Superoxide dismutase (SOD) activity was assessed using the method described by Beauchamp and Fridovich [34], . All enzyme activities were measured using an ELISA reader (BioTek Powerwave XS2). The extraction and quantification of proline were conducted based on the method by Bates et al. [35], where the proline concentration in the samples was calculated using a standard curve and the corresponding regression equation. The proline concentration in the samples was calculated using a standard curve and the corresponding regression equation. Phenolic compounds were extracted by grinding the target tissues in liquid nitrogen to prevent degradation due to moisture. Total phenolic content was determined using the Folin-Ciocalteu reagent (10%) based on the method developed by Ainsworth and Gillespie [36], . A standard curve was prepared using gallic acid solutions at concentrations ranging from 0 to 500 µg/mL under identical assay conditions.

Total phenolic content was calculated using the standard curve equation and expressed as milligrams of gallic acid equivalents per gram of dry weight (mg GAE/g DW). The total soluble carbohydrate content was measured using the method described by Wardlaw and Willenbrink [37], with slight modifications.

Malondialdehyde (MDA) content was evaluated according to the method established by Heath and Packer [38], with minor modifications. Additionally, the concentration of other aldehydes was determined using the procedure described by Meir et al. [39].

### Data analysis

A factorial analysis of variance (ANOVA) was conducted using a completely randomized design to evaluate the relative levels of gene expression. For this analysis, two biological replicates were selected, and each biological replicate was measured with two technical replicates. Additionally, variance analysis of greenhouse data, including morphological, physiological, and biochemical traits, was performed using both a factorial and a completely randomized design, with three independent replications for each treatment combination. Prior to ANOVA, the assumptions of normality and homogeneity of variance were tested using Shapiro-Wilk and Levene’s tests, respectively, in SPSS 22 software. All statistical analyses were carried out using GraphPad Prism 8, and mean comparisons among salinity levels and genotypes were performed using Duncan’s multiple range test at a significance level of *p* < 0.01.

## Supplementary Information

Supplementary Material 1.

## Data Availability

The datasets used and/or analyzed during the current study are available from the corresponding author on reasonable request.
