# Supplementary material for: Investigation of the effects of salt stress on morphological, physiological, biochemical, antioxidant characteristics, and gene expression responses in pistachio(Pistacia vera L.)
Source: BMC Plant Biol. 2026 Feb 25;26:589. doi: 10.1186/s12870-026-08408-x (PMC13041410; doi:10.1186/s12870-026-08408-x)
Supplement: Supplementary file 1 — Supplementary Material 1. [file 12870_2026_8408_MOESM1_ESM.docx]

**Table S1.**

Analysis of variance (ANOVA) of gene expression under different durations and levels of salt stress.

| Source | df | Mean Square | | | |
| --- | --- | --- | --- | --- | --- |
|  |  | *NHX1* | *Dehydrin* | *CAT* | *DREB2* |
| Salt Stress | 2 | 0.698** | 0.123** | 4.318** | 4.294** |
| Duration of Salt Stress | 1 | 0.341** | 0.153** | 2.712** | 3.807** |
| Salt Stress × Duration of Salt Stress | 2 | 0.108** | 0.033* | 1.073** | 0.929** |
| Error | 18 | .004 | .008 | .065 | .040 |

*and ** denote significance at the 5% and 1% probability levels, respectively, and ns indicates non-significance.

**Table S2.**

Analysis of variance for the traits studied under different durations and levels of salt stress.

| Source | df | Mean Square | | | | | | | | |
| --- | --- | --- | --- | --- | --- | --- | --- | --- | --- | --- |
|  |  | Catalase | Malondialdehyde | Superoxide dismutase | Chlorophyll a | Chlorophyll b | Carotenoid | Proline | Total phenolic content | Ascorbate peroxidase |
| Salt Stress | 2 | 0.000503** | 0.682** | 8.492** | 1.449** | 0.0690** | 0.1653** | 29.766** | 131.852** | 16.300** |
| Duration of Salt Stress | 1 | 0.000270** | 0.182** | 2.824** | 0.373** | 0.0270* | 0.0304* | 10.3571** | 21.125** | 5.677** |
| Salt Stress × Duration of Salt Stress | 2 | 0.000166** | 0.0431** | 0.496* | 0.192* | 0.0119^ns^ | 0.0307* | 2.665** | 12.471** | 0.820** |
| Error | 12 | 0.000001 | .009 | .118 | .017 | .005 | .006 | .111 | 1.429 | .095 |

*and ** denote significance at the 5% and 1% probability levels, respectively, and ^ns^ indicates non-significance.

**Continuation of Table S2.**

Analysis of variance for the traits studied under different durations and levels of salt stress.

| Source | df | Mean Square | | | | | | | | |
| --- | --- | --- | --- | --- | --- | --- | --- | --- | --- | --- |
|  |  | Polyphenol oxidase | Guaiacol peroxidase | Other aldehydes | Hydrogen peroxidease | Soluble carbohydrates | Root fresh weight | Root dry weight | Stem fresh weight | Stem dry weight |
| Salt Stress | 2 | 10.230** | 0.00228** | 0.131** | 1.0269** | 125.168** | 0.4367** | 0.259** | 0.651** | 0.208** |
| Duration of Salt Stress | 1 | 4.564** | 0.00115** | 0.0352** | 0.245** | 29.144** | 0.152* | 0.0505** | 0.00728^ns^ | 0.0281^ns^ |
| Salt Stress × Duration of Salt Stress | 2 | 0.972** | 0.000393* | 0.00740** | 0.0547^ns^ | 6.980** | 0.112* | 0.106** | 0.0145^ns^ | 0.025^ns^ |
| Error | 12 | .059 | 0.000085 | .001 | .015 | .158 | .023 | .004 | .005 | .008 |

*and ** denote significance at the 5% and 1% probability levels, respectively, and ^ns^ indicates non-significance.
